# Unravelling the biological divergence of CD30^+^ lymphoproliferative disorders with skin involvement: from clinical risk to precision oncology

**DOI:** 10.1016/j.ebiom.2026.106543

**Published:** 2026-10-30

**Authors:** Yi Jiang, Yang Wang

**Affiliations:** a Department of Dermatology and Venereology, Peking University First Hospital, National Clinical Research Center for Skin and Sexually Transmitted Diseases, Beijing Key Laboratory of Innovative Clinical Research and Translation in Skin Diseases, Beijing, 100034, China; b Department of Dermatology, The First Affiliated Hospital, Zhejiang University School of Medicine, Hangzhou, 310003, China; c Peking-Tsinghua Center for Life Sciences, Peking University, Beijing, 100034, China

CD30^+^ T cell lymphoproliferative disorders with cutaneous involvement (CD30^+^ T LPDs) constitute a highly heterogeneous spectrum of diseases that share overlapping histopathologic features. While lymphomatoid papulosis (LyP) represents an indolent, self-resolving entity and nodal anaplastic large cell lymphoma (nALCL) with skin involvement is uniformly aggressive, the remaining entities exhibit divergent clinical outcomes, underscoring the need to define the biological drivers of disease heterogeneity. One example is CD30^+^ mycosis fungoides (MF), which ranges from cases successfully managed with skin-directed therapies to those requiring intensive systemic interventions either early in the disease course or later upon histologic transformation (tMF).^1^ In current clinical practice, this pronounced clinical and morphological overlap frequently forces a vague diagnosis of CD30^+^ T-LPD not otherwise specified (NOS). Furthermore, while CD30 expression serves as the diagnostic hallmark and a therapeutic target for brentuximab vedotin, it remains an unreliable prognostic biomarker that cannot guide clinical risk stratification or predict treatment responses.^2^ Consequently, clinicians have long relied on clinical severity—such as lesion extent, lymphadenopathy, and systemic symptoms—to guide aggressive interventions. However, prior studies have failed to integrate the molecular landscape of CD30^+^ T LPDs with clinical risk stratification within large cohorts.^3^, ^4^

In a recent issue of eBioMedicine, *Biral* and colleagues addressed this gap by evaluating one of the largest multi-institutional genomic and transcriptomic cohort of CD30^+^ T-LPDs with skin involvement to date.^5^ In this retrospective study of 79 patients, DNA (n = 38) and RNA (n = 44) sequencing were performed on skin biopsy samples to comprehensively map the molecular landscape. By leveraging clinical records from 74 patients, the authors developed a risk-stratification model that assigned individuals into low- and high-risk groups based on real-world systemic treatment requirements, avoiding the diagnostic ambiguity caused by overlapping morphology. They validated the significant survival difference between the two groups, with a median overall survival of 24.4 years in the low-risk group versus 7.21 years in the high-risk group (*P* = 0.001).

The study shows a contrast between the genomic and transcriptomic landscapes. Despite sharing common mutational signatures (e.g., UV mutational signature, 5-methylcytosine deamination) and core genetic drivers (e.g., *KDR*, *TET2*), the two risk groups exhibited a marked biological divergence at the gene expression and immune microenvironment levels. High-risk cases uniquely harboured four recurrent or potentially oncogenic fusions and a hyperproliferative, inflammatory phenotype driven by the upregulation of cell cycle progression, DNA damage response, MYC targets, and immune activation. Conversely, low-risk cases featured abundant resting dendritic cells, reflecting a less inflammatory microenvironment—a signature that persisted even after excluding polar entities like LyP and nALCL.

This work moves the field from morphological description toward biologically informed, risk-stratified care, with implications for both clinical practice and laboratory research. For clinicians, it identifies six overexpressed, targetable genes (*IL2RA*, *BIRC3*, *PDE4D*, *CUL1*, *PRKDC*, *POLR2B*) in the high-risk group, while the presence of DNA mismatch repair deficiency and POLE mutation-associated signatures across both groups highlights potential sensitivity to immune checkpoint inhibitors.^6^, ^7^ For basic researchers, the high, shared prevalence of mutations in epigenetic modifiers (47%) and intercellular signalling cascades (50%) suggests that clinical aggressiveness is associated with transcriptional reprogramming, providing a framework to explore how chromatin remodelling cooperates with downstream pathways to drive clonal evolution.

Regarding the limitations, the retrospective risk stratification based on treatment recommendations introduces potential confounding bias and patient misclassification stemming from patient-specific factors such as age and comorbidities. Biologically, the downstream mechanisms of the novel fusion genes (such as *PRMT1::TYK2*) and upregulated pathways remain unproven. Moreover, the spatial distribution and cellular crosstalk of resting dendritic cells in indolent lesions have yet to be fully elucidated. Consequently, future prospective studies utilizing standardized and objective criteria, combined with *in vitro* functional assays and multiplex spatial profiling, are required to validate these prognostic signatures.

The classification of lymphoid malignancies is moving from broad histomorphological and basic immunophenotypic categorizations toward high-resolution molecular and genomic subtyping. While precision genomics and transcriptomics have rapidly reorganised the classification of diffuse large B-cell lymphoma and peripheral T-cell lymphoma into distinct, actionable subtypes,^8^, ^9^ a parallel consensus for molecular subtyping in cutaneous T-cell lymphoma is still lacking. In cutaneous T-cell lymphoma, molecular abnormalities are plentiful, but a stable, clinically validated taxonomy is missing—a consequence of the disease's biological complexity, clinical heterogeneity, and incompletely understood mechanisms. Although this study is a significant step forward, validated molecular subtypes of CD30+ T-LPDs are still needed before genomic findings can guide treatment choice and improve outcomes in refractory disease.

## Contributors

Y.J. drafted the original manuscript. Y.W. contributed to conceptualisation and critical revision of the manuscript. All the authors read and approved the final manuscript.

## Declaration of interests

The authors declare no conflict of interest.
